# Major Dietary Patterns Relationship with Severity of Coronary Artery Disease in Gaza-Strip, Palestine: A Cross-Sectional Study

**DOI:** 10.4314/ejhs.v31i3.17

**Published:** 2021-05

**Authors:** Mohamed Kuhail, Sakineh Shab-Bidar, Mehdi Yaseri, Kurosh Djafarian

**Affiliations:** 1 Department of Clinical Nutrition, School of Nutritional Sciences and Dietetics, Tehran University of Medical Sciences, International-Campus. Tehran, Iran; 2 Department of Community Nutrition, School of Nutritional, School of Nutritional Sciences and Dietetics, International College, Tehran University of Medical Sciences, Tehran, Iran; 3 Department of Epidemiology and Biostatistics, School of Public Health, Tehran University of Medical Sciences, Tehran, Iran

**Keywords:** Dietary patterns, Severity of coronary artery disease, Gensini score, Factor analysis, International physical activity questionnaire

## Abstract

**Background:**

The association of dietary patterns (DPs) linked to the severity of coronary artery disease (CAD) is little known. Therefore, this study aims to explore the relationship between major DPs and the severity of CAD.

**Methods:**

This cross-sectional study was conducted among423 newly discovered CAD patients (both genders, aged 35–65 years), who underwent coronary angiography. The severity of CAD was assessed by the Gensini score. All patients were tested using a semi-quantitative food frequency questionnaire and other related data through face-to-face interviews. Factor analysis and logistic regression were applied by using SPSS version-24.

**Results:**

By principal component analysis, two major DPs were identified: “Unhealthy” DP that characterized mainly by high intakes of sugar and sweets, soft drinks, salts, cooking oils, and processed meats, and “Healthy” DP that consisting high intakes of fruits, fish, poultry, vegetables, whole grains. After adjustment for confounding variables, the odds of severe CAD was significantly higher in the third (T3) and second (T2) tertile of the unhealthy pattern by 4.79 and 2.48 times more compared to the first tertile (T1) (OR 4.79; 95%CI 2.60, 8.83; P<0.001) and (OR 2.48; 95%CI 1.40, 4.39; P=0.002), respectively. However, the odds of CAD severity in the T3 and T2 of the healthy pattern was lowered by 0.24 and 0.38 times less compared to the T1 (OR 0.24; 95%CI 0.12, 0.47; P=0.002) and (OR 0.38; 95%CI 0.20, 0.73; P=0.006), respectively.

**Conclusion:**

The severity of CAD was significantly increased by the unhealthy dietary pattern, while decreased by adherence to the healthy pattern.

## Introduction

Despite a significant advances in the field of prophylaxis and treatment of cardiovascular diseases, coronary artery disease (CAD) is still the major leading cause of premature death globally including Palestine, counted about one-third of all deaths in people older than 35 years and 40%, respectively (1,2).

The accumulating evidence has shown that dietary patterns (DPs) as Mediterranean diet (MedDiet) and dietary approaches to stop hypertension (DASH) have been associated with reducing the risk of cardiovascular disease by10 to 60% (3). A few years ago, the analysis of the dietary pattern becomes a more recognizable approach to investigate diet-disease associations (4). Moreover, it is considered that the overall food complexity and can simply recognize the healthy nutritional recommendations for the general population (5). In real life, people do not take nutrients or foods alone but consume meals containing combinations of many nutrients and foods that possibly interact with each other (6). In this context, although some foods or nutrients intakes have been separately studied in relationship with CAD risk (7), the association of long-term prognosis of CAD severity and dietary patterns is little investigated (8,9).

The main cause of CAD in Gaza-Strip is an atherosclerotic disease, which resulting from hypertension, diabetes mellitus, tobacco smoking, and dyslipidemia (1,10). The unhealthy DP is one modifiable risk factor for CAD, together with the established risk factors including hypertension, dyslipidemia, hyperglycemia, and obesity/overweight are increasing the risk of CAD (10). However, the interrelationship between unhealthy DP and the attributive metabolic risk factors on the severity of CAD has not been definitely confirmed. Therefore, this study aimed to investigate the relationship between major dietary patterns and the severity of CAD among newly discovered patients with CAD in Gaza-strip, Palestine.

## Methods and Participants

**Study population and design**: A cross-sectional study was conducted among 423 newly discovered CAD patients, both genders, aged between 35 and 65 who presented with chest pain and underwent coronary angiography with a confirmed diagnosis of CAD by two interventional cardiologists from May 2018 to July 2019. However, patients with normal coronary angiography and well-known CAD (Post-Stent or Post-CABG), renal disease, hepatic dysfunction, anemia or blood disorders, familial hyperlipidemia were excluded from the study.

**Assessment of CAD severity**: The severity of CAD was assessed by the Gensini score (11,12). We calculated this score based on coronary angiography results, which determine the possible numbers and degrees of coronary artery occlusion. According to the Gensini scoring system, the severity score to each coronary artery stenosis pointed as follows: 1 point for ≤ 25%, 2 points for 26 to 50%, 4 points for 51 to 75%, 8 points for 76 to 90%, 16 points for 91 to 99%, and 32 points for total occlusion. Then, the point score was multiplied by a factor of the lesion's position in the coronary circulation: 5 for the left main coronary artery, 2.5 for the proximal of the left anterior descending coronary artery, 2.5 for the proximal of the circumflex artery, 1.5 for the mid-coronary of the left anterior descending artery, 1.0 for the right coronary artery, the distal of the left anterior descending artery, the posterolateral artery, and the obtuse marginal artery, and 0.5 for other segments. The summation of these scores was the total Gensini score. Patients were categorized as severe and non-severe CAD, in which the severe CAD was defined as 20 scores or more (11).

**Assessment of dietary patterns**: Dietary intakes were assessed by a semi-quantitative food frequency questionnaire (FFQ) which developed and validated for the Palestinian population (13). The data was collected by trained qualified nurses. All patients were asked to estimate the frequency of 98-food items consumption per day, week, or monthly basis through a face-to-face interview-based questionnaire. Moreover, all patients were shown the household measures including cups, spoons, and rulers, which were helped them to estimate the portion size in practice. The answers are seven categories as following; never, 1–3 times/month, 1–2 times/week, 3–4 times/week, 5–6 times/week, one time/day, or 2–3 times/day. DPs were extracted by using exploratory factor analysis after aggregating the food items into 27 groups according to their similarity in the nutrient contents after converting them into gram/day (see Table 1 and 3).

**Table 1:** Food groupings used in the dietary pattern analysis

Food Groups	Food Items
Low fat milk	Skim milk; Skimmed milk powder; Yogurt
Full fat milk	Whole milk; Condensed milk / Milk powder; Cottage cheese curd or fresh white cheese
Creams and cream substitutes	Cream cheese or portions; Ice Cream
Poultry	Chicken with skin; Skinless chicken
White meat	Rabbit; duck
Red meat	Beef, lamb, Cold meats
Organ meats	Beef liver, or chicken liver; Viscera: tripe, brains, other
Processed meats	Mortadella; Sausage; Hamburger
Eggs	Chicken eggs
Olives	Olives; Olive oil
Fish and shellfish	Mixed fried fish, boiled or grilled fish (sardines, tuna), salted fish, canned water fish, canned fish in oil, (oysters, clams, mussels and the like), shellfish (shrimp and the like)
Non-starchy vegetables	Cooked spinach; Cabbage, cauliflower, broccoli; Lettuce; Tomato; Onion; Eggplant, zucchini, cucumbers; Mushrooms; Canned vegetables; Garlic; Peppers; Parsley, thyme, bay leaves, oregano, cilantro, mint, Avocado; Tomato sauce, ketchup
Starchy vegetables	Carrots, pumpkin; Cooked green beans; Chips; Boiled potatoes
Whole grains	Wheat bread; Cooked cereals as bulgur and the like; Corn or canned
Refined grains	White bread; Toast bread; Cooked white rice; Pasta: macaroni, spaghetti and the like
Fruits	Lemon; Oranges, grapefruit and the like; Bananas; Apple or pear; Strawberries; Peach, apricot; Fresh figs; Watermelon, cantaloupe, pineapple; Papaya; Grapes; Mango; Guava; Kiwi fruit; Dried fruits as raisins, prunes; Fruits in syrup: peach, pear, pineapple, fig
Snacks	Bag of chips; Pizza; Pie
Sugar, sweets, and desserts	Biscuit; Croissant, pastries; Shortbread; Brownie; Chocolate powder and the like; Custard, custard pudding; Chocolate, chocolates; Jams, honey; Table Sugar; Tasty type artificial sweeteners
Coffee	Coffee; Decaffeinated coffee
Tea	Tea
Soft drinks	Soft drinks with sugar: e.g. cola, orange, lemon, Fanta etc.; Low calorie soft drinks; Fruit juice packaging
Salt and pickles	Salt; pickles
Hydrogenated fats	Margarine; Butter; Mayonnaise
Cooked oils	Corn oil; Sunflower oil
Spices	Pepper; chili
Beans and legumes	Cooked: lentils, chickpeas, black beans or white; Cooked peas
Nuts and seed products	Nuts: almonds, peanuts, hazelnuts, walnuts and the like; Tahini

**Table 3:** Factor-loading matrix for major dietary patterns

Food Groups	Major Dietary patterns	
	
	Unhealthy dietary pattern	Healthy dietary pattern
Low fat milk	-	0.410
Full fat milk	-	-
Creams and cream substitutes	0.396	-
Poultry	-	0.544
White meat	-	0.464
Red meats	0.346	-
Organ meats	-	-
Processed meats	0.476	-
Eggs	0.418	-
Olives	-	0.392
Fish and shellfish	-	0.571
Non-starchy vegetables	0.346	0.486
Starchy vegetables	0.465	0.493
Whole grains	-0.325	0.531
Refined grains	0.498	-0.380
Fruits	0.482	0.582
Snacks	-	-
Sugar, sweets, and desserts	0.688	-
Coffee	0.417	-
Tea	0.406	-
Soft drinks	0.547	-
Salt and pickles	0.517	-
Hydrogenated fats	0.315	-
Cooking-oils	0.509	-
Spices	0.494	-
Beans and legumes	-	0.496
Nuts and seed products	0.476	0.530
Variance explained (%)	15.335	27.794

Values < 0.30 were excluded for simplicity. The Kaiser Mayer Olkin measure of sampling adequacy was 0.814. Bartlett's test of Sphericity was < 0.001.

**Assessment of anthropometric, blood pressure, and metabolic syndrome**: All measurements were made by trained qualified nurses according to a standardized protocol. Bodyweight was measured using (Seca 808 digital scale; Germany) with light clothes and no shoes. Height was measured using a portable stadiometer (patients barefooted and head upright) with a measuring rod attached to the balanced Seca, Germany scale to the nearest of 0.1cm. Hip and waist circumference was measured to the nearest 0.1cm using a flexible, non-stretch Holtan tape directly on the skin at the end of a gentle expiration. Central obesity was calculated according to the WHO criteria for the Eastern-Mediterranean population, the recommended sex-specific cut-off points for waist circumference are 94 cm in men and 80 cm in women (14). Body mass index (BMI) was calculated using the standard formula of weight (in kilograms) divided by the square of height (in meters) (15). Furthermore, systolic and diastolic blood pressure (BP) were obtained from the left arm (mmHg) by a validated mercury sphygmomanometer. According to the WHO definition of hypertension, a systolic BP equal to or above 140 mmHg and/or diastolic BP equal to or above 90 mmHg, were categorized in the present study (16). Metabolic syndrome (MetS) was defined according to the international diabetes federation (IDF) criteria for the eastern Mediterranean and middle east “Arab” populations (17).

**Assessment of physical activity**: Physical activity (PA) level was evaluated by using the international physical activity questionnaire (IPAQ, short version) (18). It is a recommended tool to evaluate the PA level among adults ranging from 15 to 69 years. IPAQ estimates the weekly calorie expenditure expressed as metabolic equivalents per week (METs/wk). According to the IPAQ scoring system, patients were classified as insufficiently active (IA) <600 METs/wk, sufficiently active (SA) 600 to 1500 METs/wk; and very active (VA) ≥1500 METs/wk (19).

**Biochemical analyses**: After 12 hours' fasting, venous blood samples were collected from all patients on the day after coronary angiography by well-trained and experienced nurses. A venous blood sample (4 ml) was drawn into chemistry tubes. Blood serum was separated immediately, and the extracted serum was used to investigate fasting plasma glucose (FPG) mg/dl, total cholesterol mg/dl, high-density lipoprotein cholesterol (HDL-C) mg/dl, and triglycerides (TG) mg/dl (20). Moreover, blood chemistry analysis was done by Mindray BS-200 chemistry analyzer instrument (New-Jersey, USA).

**Statistical analysis**: All data analysis was conducted using Statistical Package for Social Science (SPSS) version 24. Food items from FFQ were converted into grams per day to quantifying the data. Then, according to the similarity of the nutrients content of each food item (21,22), we classified 98 food items from the FFQ into 27 food groups (Table 1). Principal components analysis was used to reduce a large number of detailed food-items into a smaller set of interpretable factors that have the characteristic of explaining the largest amount of variability in the specific patterns (23). The Kaiser-Mayer-Olkin coefficient test was used to test the sufficiency of the sample size for factor analysis which should be greater than 0.5, and the obtained value was 0.814 in the present study. Eigen values >2.2, the scree plot test, and interpretability were evaluated to retain factors for further analysis. An absolute factor loading ≥0.3 was used to define a subset of at least eleven food groups in each factor. The identified factors were labeled based on our interpretation of the data and on the basis of previous studies that found similar dietary patterns in adults (22,24). Participants were categorized into tertiles based on dietary pattern scores. Independent t-test, one-way ANOVA, and chisquare test were performed to assess the significant differences between quantitative and qualitative variables, respectively. Multivariable logistic regression was done to assess the magnitude of the relationship between variables by odds ratio (OR) with a 95% confidence interval (95% CI). P-values less than 0.05 were considered statistically significant.

**Ethical consideration**: The study protocol was approved by the Palestinian Health Research Council (Helsinki Ethical Committee of Research PHRC/HC/335/18) and by Ethics Committee of Tehran University of Medical Sciences (Code: IR.TUMS.VCR.REC.1397.636). Written informed consent was also obtained from each participant.

## Results

We included 423 newly discovered CAD patients. The severity of CAD was categorized as severe and non-severe CAD, in which the percentage of severe CAD patients was (63.8%) higher compared to non-severe (36.2%). The characteristics of the study population by the severity of CAD are shown in Table 2. The mean age of the patients was 56.7 years (SD=5.9). Most of the patients were males (74.2%) and about two-thirds of them were having severe CAD. The majority of the patients were married (91.5%), and 66.4% of them were found to have severe CAD. Moreover, 71.2% of the patients had only school education. Our results showed that 71.6% of the patients were under the poverty line ≤ 550$. The majority (96.5%) of the patients were did not used dietary supplements, and 65% of them had severe CAD. In addition, 56% of all patients had a family history of CAD, and 67.1% of them were having severe CAD. Almost half 51.5% of the patients were current smokers and 76.1% of them had severe CAD. The percentage of patients who had a MetS was 60.8%, and 67.7% of them had severe CAD. About 57.9% of the patients had a history of hypertension and about 59.6% of them had severe CAD. Almost half (51.3%) of the patients had systolic BP more than 140 mmHg, and about two-thirds (74.7%) of them had severe CAD. Additionally, 48.7% of patients had a history of DM, and 64.6% of them were categorized as having severe CAD. Moreover, 45.2% and 46.5% of all participants were overweight and obese, respectively; and the percentage of obese individuals was much higher (74.1%) among severe CAD cases compared to non-severe (25.9%). The mean WC was greater among severe CAD compared to the non-severe group. In addition, no significant differences between the means of fasting blood glucose and lipid profile between severe and non-severe CAD. Our results revealed that the gender, marital status, educational level, using dietary supplements, smoking, MetS, history of hypertension, having systolic BP ≥ 140 mm/Hg, BMI, waist circumference, and physical activity were significantly different between severe and non-severe CAD (P-value < 0.05 for all).

**Table 2:** Characteristics of the study population by the severity of CAD

Variables		CAD	Severe CAD	Non-Severe CAD	P value
			
		No. (%) 423 (100)	No. (%) 270 (63.8)	No. (%) 153 (36.2)	
Age	Mean ± SD	56.7 ± 5.9	57 ± 5.9	56 ± 6	0.111
Gender	Male	314 (74.2)	234 (74.5)	80 (25.5)	0.001
	Female	109 (25.8)	36 (33)	73 (67)	
Marital status	Married	387 (91.5)	257 (66.4)	130 (33.6)	0.001
	Unmarried	36 (8.5)	13 (36.1)	23 (63.9)	
Educational level	School edu.	301 (71.2)	179 (59.5)	112 (40.5)	0.004
	University edu	122 (28.8)	91 (74.6)	31 (25.4)	
Monthly income	≤ 550$	303 (71.6)	192 (63.4)	111 (36.6)	0.823
	> 550$	120 (28.4)	78 (65)	42 (35)	
Use dietary Supplements	Yes	15 (3.5)	5 (33.3)	10 (66.7)	0.025
	No	408 (96.5)	265 (65)	143 (35)	
Family history of CAD	Yes	237 (56)	159 (67.1)	78 (32.9)	0.115
	No	186 (44)	111 (59.7)	75 (40.3)	
History of current smoking	Yes	218 (51.5)	166 (76.1)	52 (23.9)	0.001
	No	205 (48.5)	104 (50.7)	101 (49.3)	
Metabolic Syndrome	Yes	257 (60.8)	174 (67.7)	83 (32.3)	0.049
	No	166 (39.2)	62 (57.8)	70 (42.2)	
History of HTN	Yes	245 (57.9)	146 (59.6)	99 (40.4)	0.040
	No	178 (42.1)	124 (69.7)	54 (30.3)	
Systolic BP (mm/Hg)	≥ 140	217 (51.3)	162 (74.7)	55 (25.3)	0.001
	< 140	206 (48.7)	108 (52.4)	98 (47.6)	
Duration of HTN (year)	Mean ± SD	8.67 ± 4.56	9.01 ± 4.38	8.17 ± 4.79	0.156
History of DM	Yes	206 (48.7)	133 (64.6)	73 (35.4)	0.763
	No	217 (51.3)	137 (63.1)	80 (36.9)	
Duration of DM (year)	Mean ± SD	8.41 ± 4.99	8.61 ± 4.98	8.06 ± 5.01	0.481
BMI (kg/m^2^)	Normal	35 (8.3)	20 (57.1)	15 (42.9)	0.001
	Overweight	191 (45.2)	104 (54.5)	87 (45.5)	
	Obese	197 (46.5)	146 (74.1)	51 (25.9)	
Waist circumference/cm	Mean ± SD	108.5 ± 11	110.1 ± 11.1	105.7 ± 10.4	0.001
FBG (mg/dl)	Mean ± SD	122.3 ± 44.7	124.3 ± 45.3	118.6 ± 43.7	0.204
Hip circumference (cm)	Mean ± SD	104.5 ± 9.6	104.8 ± 9.3	103.9 ± 10.1	0.368
Cholesterol (mg/dl)	Mean ± SD	179.3 ± 40.8	181.1 ± 41.8	167.2 ± 38.2	0.200
Triglyceride (mg/dl)	Mean ± SD	163.4 ± 59.6	166.4 ± 64.5	161.7 ± 56.6	0.439
LDL-C (mg/dl)	Mean ± SD	105.1 ± 38.8	107.5 ± 38.9	100.5 ± 38.5	0.067
HDL-C (mg/dl)	Mean ± SD	41.4 ± 6.2	41.28 ± 5.8	41.6 ± 6.6	0.587
Physical activity level (Total MET/wk)	IA ^(a)^	93 (22)	58 (62.4)	35 (37.6)	0.001
	SA ^(b)^	187 (44.2)	139 (74.3)	48 (25.7)	
	VA ^(c)^	143 (33.8)	73 (51)	70 (49)	

CAD: coronary artery disease; BP: blood pressure; HTN: hypertension; DM: diabetes mellitus; BMI: body mass index; FBG: fasting blood glucose; HDL: low-density lipoprotein; LDL: low-density lipoprotein; IA: insufficiently active (< 600 MET/wk); SA: sufficiently active (600 to 1500 MET/wk); VA: very active (≥ 1500 MET/wk); SD: stander deviation.

The factor loading matrixes for the two major dietary patterns are shown in Table 3. Based on eigenvalues we identified two major DPs: 1) Unhealthy DP consisting of thirteen food groups, which characterized by high intakes of sugar, sweets, and desserts, cooking-oils, soft drinks, salt and pickles, creams and cream substitutes, eggs, spices, refined grains, processed meats, red meats, hydrogenated fats, tea, and coffee. 2) Healthy DP consisting of eleven food groups characterized by a high intake of whole grains, fruits, nuts and seed products, starchy and non-starchy vegetables, fish and shellfish, poultry, white meats, low-fat milk, beans and legumes, and olives. While the remaining three food groups: full-fat milk, organ meats, snacks were excluded, due to low correlation coefficients r > 0.3. The two major DPs explained 15.33% and 27.79% of the total variance, respectively.

In this study, the obtained DPs scores were categorized as tertiles. Then the characteristics of the study population were evaluated within the tertiles (Table 4). Regarding the relationship between unhealthy DP tertiles and the predicted variables. The results showed that the mean age of the patients at the first tertile (T1) of the unhealthy DP was significantly higher compared to those in the second and third tertiles (T3) (P=0.021). Where the proportion of males in the middle tertile (T2) was higher compared to those in the T1 and T3 (*P*<0.001). Moreover, the history of current smoking was higher in the T2 compared to other tertiles (P=0.002). The history of hypertension was found higher in the T1 compared to the T2 and T3 (P=0.034). Additionally, the means of both blood cholesterol and LDL-C level were higher in the T2 against other tertiles (P=0.050). However, the distribution of marital status, education level, monthly income, using dietary supplements, family history of CAD, MetS, duration of hypertension and diabetes, BMI, waist circumference, and level of PA were did not reached a significant level between the unhealthy DP tertiles.

**Table 4:** Characteristics and dietary intakes of study population by tertiles (T) categories of dietary pattern scores

Characteristics	Unhealthy pattern			P-value	Healthy pattern			P-value
	
	T1	T2	T3		T1	T2	T3	
Age (year)								
Mean ± SD	57.77±5.69	56.30±5.65	55.90±6.33	0.021	56.18±6.501	56.85±5.772	56.94±5.516	0.502
Gender %								
Males	27.4	37.6	35	0.001	42.7	37.6	19.7	0.001
Females	55.5	21.1	28.4		6.4	21.1	72.5	
Marital status %								
Married	31.8	34.1	34.1	0.085	35.1	34.9	30.0	0.001
Unmarried	50.0	25.0	25.0		13.9	16.7	69.4	
Educational level %								
School Edu.	34.9	31.9	33.2	0.495	31.5	32.6	35.9	0.202
University Edu.	29.5	36.9	33.6		37.7	35.2	27.1	
Monthly income %								
≤ 2000 (NIS)	32.0	35.3	32.7	0.376	36.6	30.4	33.0	0.042
>2000 (NIS)	36.7	28.3	35		25.0	40.8	34.2	
Used dietary supplements %								
Yes	40.0	20.0	40.0	0.537	0.00	33.3	66.7	0.006
No	33.1	33.8	33.1		34.6	33.3	32.1	
Family history of CAD %								
Yes	29.5	34.6	35.9	0.163	35.9	33.3	30.8	0.353
No	38.2	31.7	30.1		30.1	33.3	36.6	
History of current smoking %								
Yes	25.7	39.0	35.3	0.002	46.8	35.8	17.4	0.001
No	41.5	27.3	31.2		19.0	30.7	50.3	
Metabolic syndrome %								
Yes	35.8	32.7	31.5	0.382	31.1	37.4	31.5	0.092
No	29.5	34.3	36.2		36.7	27.1	36.2	
History of HTN %								
Yes	38.4	31.4	30.2	0.034	31.4	31.8	36.8	0.218
No	26.4	36.0	37.6	36.0	35.4	28.6	
Systolic BP (mm/Hg) %								
< 140	36.7	33.3	30.0	0.461	33.3	26.7	40.0	0.045
≥ 140	31.5	33.3	35.2		33.3	37.0	29.7	
Duration of HTN (year)								
Mean ± SD	8.71 ±4.26	8.46 ±4.81	8.85 ±4.71	0.867	8.56 ±4.48	8.32 ±3.65	9.09 ±5.30	0.529
History of DM %								
Yes	37.9	33.5	28.6	0.077	30.1	32.5	37.4	0.191
No	29.0	33.2	37.8		36.4	34.1	29.5	
Duration of DM (year) %								
Mean ± SD	9.94 ±5.79	9.12 ±5.97	9.02 ±6.76	0.622	9.15 ±6.98	9.49 ±5.82	9.52 ±5.73	0.930
BMI (kg/m2) %								
Normal	31.4	25.7	42.9	0.117	31.4	22.9	45.7	0.269
Overweight	39.3	31.4	29.3		30.9	33.5	35.6	
Obesity	27.9	36.5	35.5		36.0	35.0	29.0	
Waist circumference (cm)								
Mean ± SD	108.1±9.87	107.74±10.95	109.72±12.07	0.285	108.84 ± 10.29	110.08±10.07	106.70 ± 12.33	0.033
FBG (mg/dl)								
Mean ± SD	126.28±44.85	125.68 ± 49.06	114.83 ± 39.11	0.053	117.79 42.19	122.72 ± 42.03	126.27 ± 49.49	0.280
Cholesterol (mg/dl)								
Mean ± SD	171.75 ± 37.84	185.77 ± 43.36	180.50 ± 40.26	0.014	182.50 ± 37.47	179.92 ± 42.44	175.59 ± 42.46	0.358
Triglyceride (mg/dl)								
Mean ± SD	167.62 ± 62.31	167.63 ± 62.32	160.23 ± 60.74	0.563	162.21 ± 57.76	160.98 ± 52.09	166.97 ± 68.00	0.673
HDL-Cholesterol (mg/dl)								
Mean ± SD	41.07 ± 6.93	41.75 ± 6.07	41.39 ± 5.38	0.651	41.44 ± 6.09	41.11 ± 6.25	41.66 ± 6.15	0.756
LDL- Cholesterol (mg/dl)								
Mean ± SD	97.05 ± 35.79	112.01 ± 40.27	106.29 ± 38.99	0.005	108.69 ± 33.25	106.43 ± 40.50	100.23 ± 41.88	0.166
Physical activity level (Total MET/wk) %								
IA	40.9	34.4	24.7	0.296	26.9	36.6	36.5	0.001
SA	31.5	33.7	34.8		34.8	41.7	23.5	
VA	30.7	32.2	37.1		35.7	20.3	44.1	

CAD: coronary artery disease; BP: blood pressure; HTN: hypertension; DM: diabetes mellitus; BMI: body mass index; FBG: fasting blood glucose; HDL: low-density lipoprotein; LDL: low-density lipoprotein; IA: insufficiently active (< 600 MET/wk); SA: sufficiently active (600 to 1500 MET/wk); VA: very active (≥ 1500 MET/wk). SD: stander deviation

On the other hand, the characteristics of the study population across healthy DP tertiles are presented in Table 4. The results showed that the proportion of male, married, low monthly income ≤ 550$, and current smoking was higher in the T1 compared to T2 and T3 (P<0.05 for all). Moreover, the proportion of patients using dietary supplements, had a low systolic BP less than 140 mm/Hg, had a low mean of waist circumference, and being very physically active were higher in the T3 compared to the T1 and T2 (P<0.05 for all). Furthermore, the proportion of insufficient PA< 600 MET/wk was higher in T2 and T3 compared to the first one (*P*<0.001). However, our findings showed that no significant differences were found between healthy pattern tertiles and the following factors including age, education level, family history of CAD, MetS, history of hypertension and diabetes, BMI, and blood lipid profile.

Finally, the OR and corresponding 95% CIs for CAD severity across tertiles of major DPs scores are presented in Table 5. Our results indicated that in the crude model, the odds ratio of severe CAD in the T2 of unhealthy DP was significantly higher by 3 times more compared to the first tertile (OR 3; 95% CI 1.83, 4.90; P<0.001). Likewise, the severity of CAD was significantly higher by 2.48 times more among the T3 of unhealthy DP against reference tertile (OR 2.48; 95% CI 1.40, 4.39; P=0.002). Regarding the association of severity of CAD and healthy DP. The analysis showed that the unadjusted odds of the severe CAD in the T2 of the healthy DP was lower by 0.44 times less compared to T1 (OR 0.44; 95% CI 0.25, 0.78; P=0.005). Moreover, the severity of CAD in the T3 of healthy DP was decreasing by 0.38 times compared to T1 (OR 0.38; 95% CI 0.20, 0.73; P=0.006). However, after adjustment for confounding variables age, gender, marital status, history of hypertension, current smoking, duration of diabetes, waist circumference, and HDL-C, the odds of severe CAD was increased by 4.34 times more among the T2of unhealthy DP compared to the T1 group (OR 4.34; 95% CI 2.59, 7.26; *P*<0.001). Additionally, the odds ratio of severe CAD in the T3 of unhealthy DP was also increased by 4.79 times more compared to the referral group (OR 4.79; 95% CI 2.60, 8.83; *P*<0.001). Furthermore, the odds of CAD severity in the T2 of healthy DP was decreased by 0.12 times less compared to T1 (OR 0.12; 95% CI 0.06, 0.21; *P*<0.001). Moreover, the odds of severe CAD was decreased by 0.24 times among T2 of healthy DP compared to the T1 group (OR 0.24; 95% CI 0.12, 0.47; P=0.002).

**Table 5:** Logistic regression of CAD severity across tertiles (T) of unhealthy and healthy dietary patterns

CAD severity (non-severe/ Severe CAD)	Unhealthy dietary pattern				

	T1	T2		T3	
	Ref.	OR (CI 95%)	P-value	OR (CI 95%)	P-value
No.	79/62	42/99		32/109	
Crude	1	3.00 (1.83–4.90)	0.001	4.34 (2.59–7.26)	0.001
Adjusted*	1	2.48 (1.40–4.39)	0.002	4.79 (2.60–8.83)	0.001

	Healthy dietary pattern				
No,	23/118	43/98		87/54	
Crude	1	0.44 (0.25–0.78)	0.005	0.12 (0.06–0.21)	0.001
Adjusted**	1	0.38 (0.20–0.73)	0.006	0.24 (0.12–0.47)	0.002

* Adjusted for age, gender, marital status, history of hypertension, currant smoking, family history of CAD, waist circumference, LDL-Cholesterol, Cholesterol, glucose level, metabolic syndrome.

** Adjusted for age, gender, marital status, education years, monthly income in NIS, history of hypertension, waist circumference, history of DM, family history of CAD, currant smoking, supplementation intake, and metabolic syndrome. P-value less than 0.05 was considered as statistically significant. CAD, coronary artery disease

## Discussion

To the best of our knowledge, this is the first study that examined the dietary patterns in relationship with the severity of CAD among newly discovered patients in Gaza-strip, Palestine. By using principal components analysis, two major DPs were recognized. The results showed that the unhealthy DP, which characterized by a high intake of (sugar, sweets, and desserts), cooking-oils, soft drinks, salt and pickles, creams and cream substitutes, eggs, spices, refined grains, processed meats, red meats, hydrogenated fats, tea, and coffee was associated with a higher risk of severe CAD. According to our review of previous studies, few studies have explained the association of severity of CAD and the DPs (8,25,26). A recent meta-analysis conducted by Zhang et al (27) reported that unhealthy/Western DP, which characterized by high consumption of red and processed meats, refined grains, sweets, high-fat dairy products, butter, potatoes, high-fat gravy, and low intakes of fruits and vegetables was associated with increased risk of CAD.

Our results were alongside a recent two similar cohort work conducted by Oikonomou et al and Georgiopoulos et al (8,28) among 188 symptomatic stable CAD patients, using the Gensini score to evaluate the severity of CAD. The authors showed that the western/ unhealthy DP, which consisting of an increase in the consumption of fat, red meats, carbohydrates, and minimal consumption of fruits vegetables, and green leafy was significantly increased the risk of CAD severity and the extent of lesions. Similarly, Mahalle et al (25) recurred 300 well-known CAD patients, above the age of 25 years, and stratified their CAD severity based on the number of vessels single, double, and triple. The authors found that patients with triple vessel disease had a higher intake of carbohydrates, and fat compared to single-vessel disease. Although the researchers used a different way to evaluate the severity of CAD, the dietary factor also can predict the severity of CAD.

Consistently, the three studies also came alongside with our findings (29–31) and reported that a higher adherence to western/unhealthy DP consisting of high consumption of sweets, pasta, potatoes, red meat, and low consumption of fruits and vegetables was associated with a higher probability of incidence of severe CAD. Another study conducted by Oikonomou, et al (8) among symptomatic stable patients with CAD in Athens, Greece showed that unhealthy/western DP (increased intake of fat, carbohydrates, and red meats, and minimal consumption of fruits and green leafy vegetables) was associated with severity of coronary artery lesions. It was hypothesized that more pro-inflammatory diets would be associated with an increase in inflammatory biomarkers that leads to an increase in circulatory disorders (32).

On the other hand, the results of this study revealed that the healthy DP which is characterized by a high intake of whole grains, fruits, nuts and seed products, starchy vegetables, non-starchy vegetables, fish and shellfish, poultry, white meats, low-fat milk, beans and legumes, and olives was inversely associated with severity of CAD. Our results come consistently with the finding of the large INTERCATH cohort study on 1121 patients who were undergoing coronary angiography in Germany. The authors showed that patients who adherence to MedDiet-style which characterized by high consumption of fruits, fish, whole grains, and nuts and legumes had less probability for a medium/high risk of CAD severity on the SYNTAX score (26). Similarly, Akgüllü et al (33) conducted a study among two-hundred CAD patients in Turkey, who were assessed for a 10-point MedDiet-scale. The author showed a negative correlation between MedDiet-score and the severity of CAD.

The current scientific evidence allows recommended dietary patterns that combined multiple foods and nutrients. Because of the synergistic effects among them, such as a high intake of fruits, vegetables, fish and seafood, nuts, seeds, whole grains, olive and vegetable oils, and dairy foods are typically reflected the high intake of vitamins, minerals, fiber, antioxidants, monounsaturated, and polyunsaturated fatty acids (34,35), that might act potentially as anti-inflammatory and prevent the progress of atherosclerotic coronary plunge.

The main limitations of this study are that it is a cross-sectional design; the causal relationship could not be determined, and it limits the generalizability of the results. In addition, the possibility of recall bias and misreporting by using FFQ assessment of dietary patterns are other limitations. Describing the relationship between major dietary patterns and severity of CAD for the first time among all newly discovered CAD patients is the main strength of this study.

We conclude that the unhealthy dietary pattern was positively and significantly associated with the severity of CAD. While the healthy dietary pattern was found protective for severe CAD among newly discovered patients in Gaza-strip.
